# Vertical Sleeve Gastrectomy Reduces Gut Luminal Deoxycholic Acid Concentrations in Mice

**DOI:** 10.1007/s11695-024-07288-0

**Published:** 2024-05-22

**Authors:** Rahaf Shishani, Annie Wang, Victoria Lyo, Renu Nandakumar, Bethany P. Cummings

**Affiliations:** 1 Department of Surgery, Division of Foregut, Metabolic, and General Surgery, Center for Alimentary and Metabolic Sciences, School of Medicine, University of California - Davis, Sacramento, CA 95817 USA; 2 Department of Molecular Biosciences, School of Veterinary Medicine, University of CA – Davis, Davis, CA 95616 USA; 3 Biomarkers Core Laboratory, Irving Institute for Clinical and Translational Research, Columbia University Irving Medical Center, Columbia University, New York, NY 10032 USA

**Keywords:** Vertical sleeve gastrectomy, Deoxycholic acid, Bile acids

## Abstract

**Background:**

Bariatric surgery alters bile acid metabolism, which contributes to post-operative improvements in metabolic health. However, the mechanisms by which bariatric surgery alters bile acid metabolism are incompletely defined. In particular, the role of the gut microbiome in the effects of bariatric surgery on bile acid metabolism is incompletely understood. Therefore, we sought to define the changes in gut luminal bile acid composition after vertical sleeve gastrectomy (VSG).

**Methods:**

Bile acid profile was determined by UPLC-MS/MS in serum and gut luminal samples from VSG and sham-operated mice. Sham-operated mice were divided into two groups: one was fed ad libitum, while the other was food-restricted to match their body weight to the VSG-operated mice.

**Results:**

VSG decreased gut luminal secondary bile acids, which was driven by a decrease in gut luminal deoxycholic acid concentrations and abundance. However, gut luminal cholic acid (precursor for deoxycholic acid) concentration and abundance did not differ between groups. Therefore, the observed decrease in gut luminal deoxycholic acid abundance after VSG was not due to a reduction in substrate availability.

**Conclusion:**

VSG decreased gut luminal deoxycholic acid abundance independently of body weight, which may be driven by a decrease in gut bacterial bile acid metabolism.

**Graphical Abstract:**

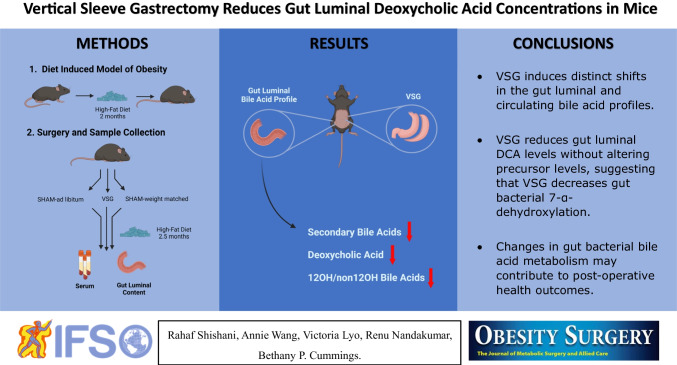

**Supplementary Information:**

The online version contains supplementary material available at 10.1007/s11695-024-07288-0.

## Introduction

Bariatric surgery remains the most effective long-term treatment for severe obesity and its associated complications, including type 2 diabetes and cardiovascular disease [1, 2]. Beyond its primary goal of weight reduction, bariatric surgery exerts profound metabolic effects by ameliorating the main components of metabolic syndrome, such as hyperglycemia, hypertension, and dyslipidemia [3–5]. While the mechanisms responsible for the remarkable post-operative improvements in metabolic health after bariatric surgery remain incompletely defined, increases in bile acid receptor signaling and improvements in bile acid metabolism have been highlighted as key contributors [6–8].

Bile acids, traditionally recognized as crucial facilitators of lipid digestion and absorption, are also key mediators of glucose homeostasis, serving as ligands for farnesoid X receptor (FXR) and the G protein-coupled bile acid receptor, TGR5 [9]. FXR regulates bile acid, glucose, and lipid homeostasis [10]. Similarly, TGR5 promotes glucose regulation and energy expenditure [11]. Work in mouse models of bariatric surgery has demonstrated that FXR and TGR5 contribute to improved post-operative glycemic improvements [11, 12]. However, the specific mechanisms by which bariatric surgery alters bile acid dynamics are incompletely defined.

The two key compartments of bile acid metabolism are the liver and the gut microbiome. The primary bile acids, cholic acid (CA) and chenodeoxycholic acid (CDCA) are made in the liver and originate from cholesterol [9]. Primary bile acids are conjugated to the amino acids, glycine or taurine, and then stored in the gallbladder and secreted into the gut. In the distal gut, bile acids undergo critical transformations, including deconjugation via bile salt hydrolase (BSH), and conversion to secondary bile acids through 7-α-dehydroxylation [9]. Specifically, the primary bile acids CA and CDCA are converted to deoxycholic acid (DCA) and lithocholic acid (LCA), respectively. Thus, the gut microbiome is directly involved in the regulation of bile acid metabolism. Conversely, bile acids regulate the growth and composition of the gut microbiome [13]. Therefore, gastrointestinal homeostasis is subject to the joint regulation of bile acids and the gut microbiome.

VSG-induced alterations in circulating bile acid profiles have been well-documented in both human and rodent studies [8, 14, 15]. However, limited information is available regarding changes in the gut luminal bile acid profile. Additionally, many studies of bariatric surgery have reported alterations in gut microbiome composition in both rodents and humans [16–18]. Gut microbiome transplantation from mice or human patients after bariatric surgery into germ-free mice has been shown to recapitulate the metabolic benefits of bariatric surgery [19, 20]. Therefore, the gut microbiome has been shown to contribute to the metabolic benefits of bariatric surgery; however, the mechanisms remain incompletely understood. Therefore, we sought to assess the impact of surgical weight loss on gut luminal bile acid profiles. We tested the hypothesis that vertical sleeve gastrectomy (VSG) induces a beneficial shift in the gut luminal bile acid profile. In this study, we report a substantial decrease in gut luminal DCA abundance following VSG in mice. Together, these data suggest that VSG alters gut bacterial bile acid metabolism which may contribute to the gut microbiome-derived effects of this operation.

## Methods

### Animals and Diet

To test our hypothesis, we studied samples collected from mice we have previously reported on [21]. All animal procedures were approved by the Cornell University Institutional Animal Care and Use Committee. Briefly, male C57BL/6J diet-induced obese mice were placed on a 60% energy from fat high-fat diet (HFD) (D12492, Research Diets) at 2 months of age and were maintained on HFD throughout the study. At 4 months of age, mice underwent sham or VSG surgery (*n* = 6). VSG and sham surgery were performed as previously described [11]. Sham-operated mice were divided into two groups: ad libitum fed (S-AL) (*n* = 5) or food restricted to match body weight to VSG-operated mice (S-WM) (*n* = 6). All mice with available cecal and serum data were included in this study. One mouse from the S-AL group was excluded from all analyses after being identified as an outlier (Grubb’s test, *P* < 0.01).

### Bile Acid Quantification

At 2.5 months after surgery, mice were fasted overnight, and a fasting tail-blood sample was collected prior to euthanasia by an overdose of pentobarbital (200 mg/kg IP). Gut luminal contents were collected from the cecum. Serum and gut luminal contents were analyzed in a blinded fashion for bile acids by UPLC-MS/MS at the Biomarkers Core Laboratory in the Irving Institute for Clinical and Translational Research at Columbia University Medical Center using Waters Xevo TQS mass spectrometer integrated with an Acquity UPLC system (Milford, MA), as previously described [22].

### Statistical Analysis

All data are expressed as mean ± SEM. All statistical analyses were performed using GraphPad Prism V9. Data were analyzed using one-factor ANOVA with Tukey’s post hoc analysis. Differences were considered significant at *P* ≤ 0.05.

## Results

### VSG Reduces Gut Luminal Secondary Bile Acid Levels

**Fig. 1:**
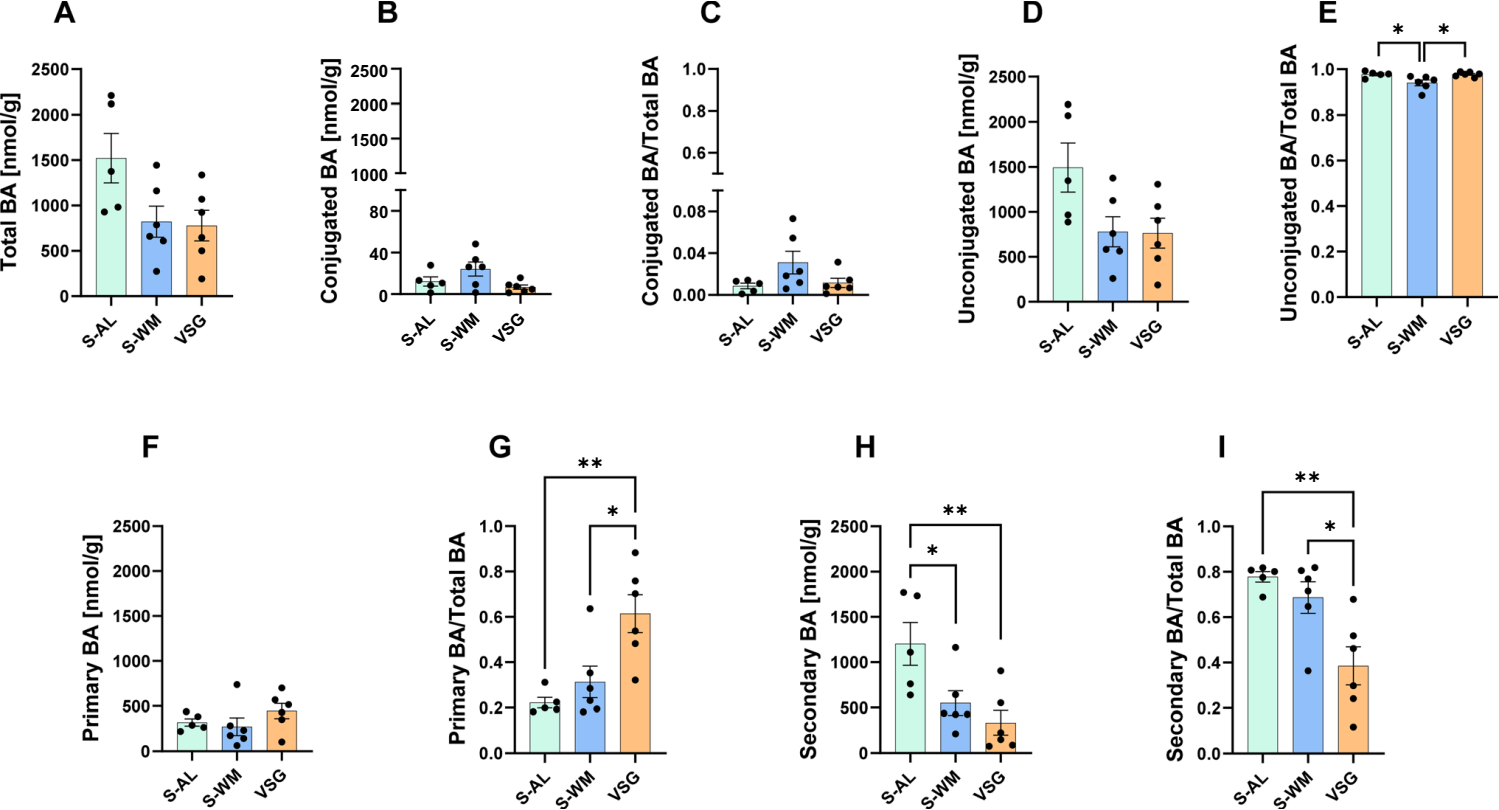
VSG reduces gut luminal secondary bile acid levels. **A** Total bile acids (BA), **B** conjugated bile acid concentration and **C** abundance, **D** unconjugated bile acid concentration and **E** abundance, **F** primary bile acid concentration and **G** abundance, **H** secondary bile acid concentration, and **I** abundance in gut luminal contents. Data presented as mean ± SEM, *n* = 5–6 per group. *P < 0.05, **P < 0.01 by one-factor ANOVA with Tukey’s post-test

To determine the impact of bariatric surgery on gut luminal bile acid profile, we studied mice after sham and VSG surgery. Sham-operated mice were either fed ad libitum (S-AL) or were food-restricted to match body weight to VSG-operated mice (S-WM). The metabolic impact of VSG in these mice has been previously reported. Briefly, VSG lowered body weight gain and improved glucose tolerance compared to sham-operated ad libitum-fed controls [21]. Total bile acid concentrations in gut luminal contents tended to be lower in VSG compared to S-AL mice (Fig. 1A, *P* = 0.056). Therefore, both absolute bile acid concentrations and the relative proportion of specific bile acids within the total gut luminal bile acid pool are presented. Detailed gut luminal bile acid concentrations and abundances are presented in Supplemental Table S1. Additionally, detailed circulating bile acid concentrations and abundances are presented in Supplemental Table S2.

To assess the potential impact of VSG on gut bacterial bile acid metabolism, we examined the effects of VSG on unconjugated and secondary bile acid levels within the gut lumen. There were no discernible differences in the concentration (Fig. 1B) or proportional abundance of conjugated gut luminal bile acids between groups (Fig. 1C). However, VSG tended to decrease the concentration of gut luminal unconjugated bile acids in comparison to S-AL (Fig. 1D). When analyzed as a proportion of total gut luminal bile acids, both S-AL and VSG increased the abundance of unconjugated bile acids when compared to the S-WM group (Fig. 1E, *P* < 0.05).

Gut luminal primary bile acid concentrations did not differ between groups (Fig. 1F). However, when normalized to total bile acids, the proportional abundance of primary bile acids increased after VSG when compared to both sham-operated groups (Fig. 1G). Consistent with the proportional lowering of primary bile acids, VSG-operated mice exhibited a reduction in the concentration of secondary bile acids in the gut lumen compared to S-AL mice (Fig. 1H, *P*  < 0.01) and in the abundance of secondary bile acids in the gut lumen compared to both S-AL and S-WM mice (Fig.1I, *P* < 0.05). These data demonstrate that VSG decreases gut luminal secondary bile acid abundance independently of body weight.

### VSG Reduces Gut Luminal DCA Levels

**Fig. 2:**
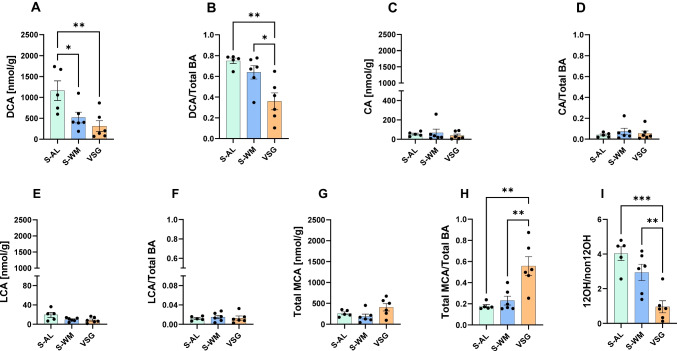
VSG reduces gut luminal DCA levels. **A** Unconjugated DCA concentration and **B** abundance, **C** unconjugated CA concentration and **D** abundance, **E** unconjugated LCA concentration and **F** abundance, **G** total MCA concentration and **H** abundance, and **I** 12α-OH/non-12α-OH ratio in gut luminal contents. Data presented as mean ± SEM, *n* = 5–6 per group. *P < 0.05, **P < 0.01, ***P < 0.001 by one-factor ANOVA with Tukey’s post-test

We next investigated what was driving the reduction in gut luminal secondary bile acid levels following VSG and found that it was primarily due to a decrease in DCA. VSG lowered gut luminal DCA concentrations compared to S-AL (Fig. 2A, *P* < 0.01) and proportionally decreased gut luminal DCA abundance compared to both S-AL and S-WM (Fig. 2B, *P* < 0.01). S-WM mice exhibited a decrease in gut luminal DCA concentration, but not abundance, compared with S-AL (Fig. 2A, B, *P* < 0.05). CA is the precursor for gut bacterial production of DCA. However, gut luminal CA concentration (Fig. 2C) and abundance (Fig. 2D) did not differ between groups. Therefore, the observed decrease in gut luminal DCA abundance after VSG was not due to a reduction in substrate availability. In contrast, gut luminal concentration, and abundance of another secondary bile acid, LCA, did not differ between groups (Fig. 2E, F). Furthermore, CDCA, the precursor to LCA, did not differ between groups (Supplemental Tables S1 and S2). Therefore, the effect of VSG to reduce gut luminal secondary bile acid levels is driven by a decrease in DCA.

The effect of VSG to cause a proportional increase in gut luminal primary bile acids was driven by a proportional increase in MCA. While gut luminal MCA concentrations did not differ between groups (Fig. 2G), there was a proportional increase in the abundance of MCA in VSG compared to S-AL and S-WM (Fig. 2H, *P* < 0.01).

Aside from its association with increased bile acid synthesis, obesity can lead to a preference for hepatic 12-α-hydroxylation. An increased ratio of 12-α hydroxylated/non-12-α-hydroxylated (12OH/non12OH) bile acids is associated with dyslipidemia and insulin resistance [23]. It has been previously found that bariatric surgery decreases this ratio in the circulation [5, 24]. Consistent with this, VSG reduced the gut luminal 12OH/12nonOH bile acid ratio compared to S-AL and S-WM (Fig. 2I, *P* < 0.01), demonstrating that VSG lowers this ratio in gut luminal contents independently of body weight.

### The Effect of VSG to Decrease Gut Luminal DCA Levels Is Not Reflected in the Circulation

**Fig. 3:**
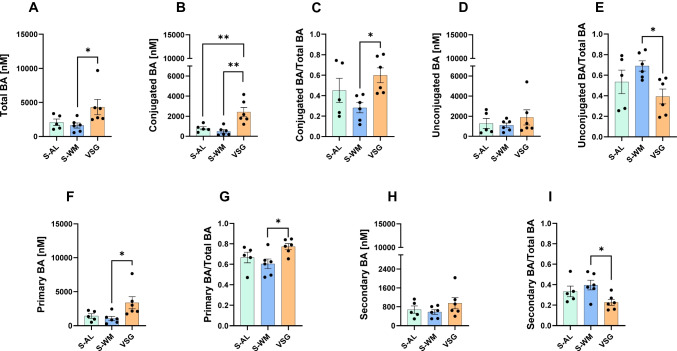
VSG increases circulating conjugated bile acid levels. **A** Total bile acids, **B** conjugated bile acid concentration and **C** abundance, **D** unconjugated bile acids concentration and **E** abundance, **F** primary bile acid concentration and **G** abundance, **H** secondary bile acid concentration, and **I** abundance in serum. Data presented as mean ± SEM, *n* = 5–6 per group. *P  ≤0.05, **P < 0.01 by one-factor ANOVA with Tukey’s post-test

In contrast to the gut luminal data, VSG increased total circulating bile acid concentrations compared to S-WM (Fig. 3A, *P* = 0.05). The elevation of total bile acid concentrations was primarily due to increased conjugated bile acids as both an absolute value (Fig. 3B, *P* < 0.01) and abundance (Fig. 3C, *P* < 0.05) compared to S-WM. Additionally, while VSG had no effect on unconjugated bile acid concentrations (Fig. 3D), VSG decreased unconjugated bile acid abundance compared to S-WM (Fig. 3E, *P* < 0.05).

Circulating primary bile acid concentrations and abundance mirrored our findings in the gut luminal pool. Specifically, VSG increased primary bile acid concentrations and abundance compared to S-WM (Fig. 3F, G, *P* < 0.05). While circulating secondary bile acid concentrations did not differ between groups (Fig. 3H), VSG decreased secondary bile acid abundance compared to S-WM (Fig. 3I, *P* < 0.05).

**Fig. 4:**
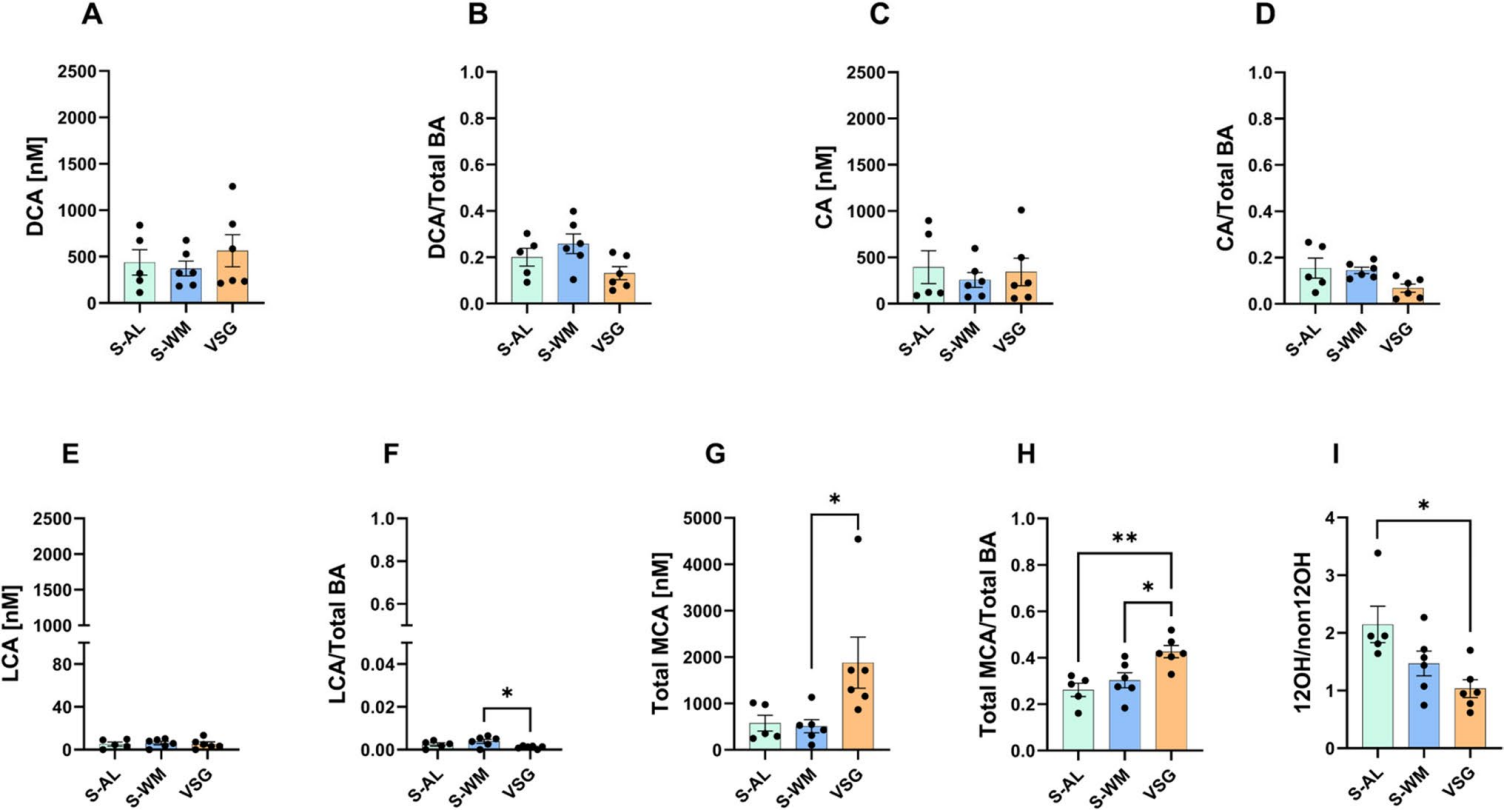
Effect of VSG to increase gut luminal DCA levels is not reflected in the circulation. **A** Unconjugated DCA concentration and **B** abundance, **C** unconjugated CA concentration and **D** abundance, **E** unconjugated LCA concentration and **F** abundance, **G **total MCA concentration and **H** abundance, and **I** 12α-OH/non-12α-OH ratio in serum. Data presented as mean ± SEM, *n* = 5–6 per group. *P < 0.05, **P < 0.01 by one-factor ANOVA with Tukey’s post-test

In contrast to the observations in the gut lumen, neither circulating DCA concentrations (Fig. 4A) nor abundance (Fig. 4B) differed between groups. Likewise, circulating CA concentration (Fig. 4C) and abundance (Fig. 4D) did not differ between groups, mirroring the findings in the gut lumen. However, while LCA concentration (Fig. 4E) remained consistent between groups, its abundance decreased after VSG when compared to S-WM (Fig. 4F, *P* < 0.05). This reduction in LCA abundance coincides with a decrease in CDCA abundance in the circulation (Supplemental Table S2, *P* < 0.01). VSG increased circulating MCA concentrations compared with S-WM and increased MCA abundance compared to both S-AL and S-WM (Fig. 4G, H, *P* < 0.05). Similar to our findings in the gut lumen, the circulating 12OH/non12OH bile acid ratio was decreased in VSG compared to S-AL mice (Fig. 4I, *P* < 0.05).

### VSG Increases Circulating Taurine-Conjugated Bile Acid Concentrations

Previous studies have reported that obesity reduces circulating conjugated bile acid concentrations, particularly taurine-conjugated bile acids [25, 26]. Additionally, individuals with obesity tend to have a lower total body content of taurine, and long-term taurine supplementation has demonstrated the potential to promote weight loss [27], implying an anti-obesity role for taurine. Furthermore, VSG [24], RYGB [25], and duodenal-jejunal bypass [28] surgeries have been reported to increase circulating concentrations of taurine-conjugated bile acids. These increased concentrations might be connected to the metabolic benefits of bariatric surgery, as taurine-conjugated bile acids have a high affinity for the TGR5 receptor [29]. We have also previously found that TGR5 signaling contributes to the metabolic improvements seen after VSG [11]. Therefore, we sought to investigate the effects of VSG on gut luminal taurine-conjugated bile acid concentrations.

**Fig. 5:**
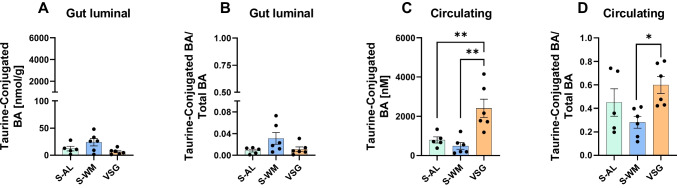
VSG increases circulating taurine-conjugated bile acid levels. **A** Gut luminal total taurine conjugated bile acid concentration and **B** abundance, and **C** circulating total taurine conjugated bile acid concentration and **D** abundance. Data presented as mean ± SEM, *n* = 5–6 per group. *P < 0.05, **P < 0.01 by one-factor ANOVA with Tukey’s post-test

While gut luminal taurine-conjugated bile acids did not differ between groups (Fig. 5A, B), our circulating bile acid data aligns with our previous findings [3] of VSG causing a substantial increase in circulating taurine-conjugated bile acid levels, both in terms of concentration (Fig. 5C, *P* < 0.01) and abundance (Fig. 5D, *P* < 0.05) when compared to S-WM. These differences in gut luminal and serum concentrations may be attributed to the fact that taurine-conjugation reduces hydrophobicity, making these bile acids more likely to stay in the serum.

## Discussion

There is growing recognition that postoperative changes in bile acid metabolism play a pivotal role in the effect of bariatric surgery to improve metabolic health. Nevertheless, our understanding of the factors that contribute to these changes in bile acid metabolism remains incomplete. In particular, there is limited information on the impact of bariatric surgery on gut luminal bile acid profiles. Herein, we report that VSG in mice lowers gut luminal secondary bile acid levels which is driven by a reduction in gut luminal DCA levels. Moreover, our serum analysis supports previous findings of a shift in the circulating bile acid composition, driven by an increase in taurine-conjugated bile acids following VSG [3].

Our findings align with observations from a study in humans that found a reduction in fecal DCA concentrations following RYGB [30]. While VSG reduced gut luminal DCA abundance, gut luminal CA abundance did not differ between groups. This suggests that the reduction in DCA abundance is not due to a decrease in substrate and, instead, may be due to a decrease in gut bacterial DCA production. The gut microbiome plays an important role in shaping the composition of the bile acid pool. Specifically, the production of DCA depends on 7-α-dehydroxylation, which is performed by gut bacteria in the distal small intestine and the colon [9].

Less than 0.1% of gut bacteria are thought to be capable of performing 7-α-dehydroxylation. Present consensus points to the *Clostridium* genus, specifically *C. scindens* and *C. hylemonae,* as the primary gut bacteria capable of performing 7-α-dehydroxylation, with varying efficiency [31]. Interestingly, we have previously found that VSG does not alter the relative abundance of the *Clostridium* genus relative to sham-operated control mice [5]. This is intriguing but not surprising, as a limited number of bacterial strains capable of performing 7-α-dehydroxylation have been isolated and characterized thus far, with recent literature suggesting that there may be other bacteria involved, either directly or indirectly [32]. Furthermore, human clinical studies have shown that the gut microbiome changes significantly after bariatric surgery, with some studies reporting decreases in Clostridia and Firmicutes [33–38]. Overall, further work is needed to understand how bariatric surgery alters 7-α-dehydroxylation.

The impact of bariatric surgery extends beyond metabolic disease, affecting conditions including inflammatory bowel disease (IBD) and colorectal cancer (CRC). Given the established roles of bile acids in the pathogenesis of IBD and CRC [39–41], alterations in gut bacterial bile acid metabolism following bariatric surgery could significantly influence the development and pathogenesis of these conditions. In particular, DCA is known for its ability to promote inflammation, kill bacteria, and trigger increased secretion in the colon [42, 43]. Therefore, the impact of bariatric surgery on gut luminal DCA concentrations likely influences various health outcomes after bariatric surgery.

Our study has limitations, particularly in translating data from mice to humans due to species differences. However, the use of mice offers enhanced experimental control and mechanistic insight. Further work is needed to confirm these findings in human patients and determine how bariatric surgery alters gut bacterial bile acid metabolism.

### Supplementary Information

Below is the link to the electronic supplementary material.

Supplementary file1 (DOCX 32 KB)
