# Supplementary material for: Vertical Sleeve Gastrectomy Reduces Gut Luminal Deoxycholic Acid Concentrations in Mice
Source: Obes Surg. 2024 May 22;34(7):2483–91. doi: 10.1007/s11695-024-07288-0 (PMC11217124; doi:10.1007/s11695-024-07288-0)
Supplement: Supplementary file 1 — Supplementary file1 (DOCX 32 KB) [file 11695_2024_7288_MOESM1_ESM.docx]

**Supplemental Tables**

**Supplemental Table S1. Gut luminal bile acid concentrations and abundances**.

| **Gut luminal bile acid concentrations (nmol/g)** | | | | | | |
| --- | --- | --- | --- | --- | --- | --- |
|  | **S-AL** | **S-WM** | **VSG** | **S-AL vs  S-WM** | **S-AL vs VSG** | **S-WM vs VSG** |
| **CA** | 54.2 ± 9.6 | 66.1 ± 39.2 | 38.4 ± 15.9 | 0.95 | 0.91 | 0.73 |
| **CDCA** | ND | 0.4 ± 0.44 | ND | - | - | - |
| **DCA** | 1162.7 ± 233.8 | 516.9 ± 128.8 | 313.7 ± 130.3 | 0.04 | <0.01 | 0.64 |
| **GCA** | 0.07 ± 0.07 | 0.04 ± 0.04 | ND | 0.84 | - | - |
| **GDCA** | ND | 0.05 ± 0.05 | 0.09 ± 0.09 | - | - | 0.88 |
| **HDCA** | 17.8 ± 4.5 | 20.4 ± 5.0 | 8.4 ± 2.8 | 0.91 | 0.30 | 0.13 |
| **LCA** | 19.3 ± 5.2 | 9.6 ± 1.5 | 8.5 ± 2.4 | 0.12 | 0.07 | 0.96 |
| **α-MCA** | 174.5 ± 26.4 | 123.3 ± 38.6 | 263.8 ± 61.6 | 0.73 | 0.40 | 0.11 |
| **β-MCA** | 80.8 ± 8.9 | 60.6 ± 20.3 | 137.2 ± 27.6 | 0.80 | 0.20 | 0.05 |
| **TCA** | 5.2 ± 2.4 | 7.3 ± 2.2 | 1.5 ± 0.6 | 0.73 | 0.36 | 0.09 |
| **TCDCA** | 0.1 ± 0.1 | 0.4 ± 0.2 | ND | 0.45 | - | - |
| **TDCA** | 1.3 ± 0.5 | 2.0 ± 1.2 | 0.5 ± 0.3 | 0.80 | 0.74 | 0.34 |
| **TMCA (α+β)** | 5.0 ± 1.6 | 13.7 ± 5.5 | 4.4 ± 1.4 | 0.24 | 0.99 | 0.18 |
| **TUDCA** | 0.6 ± 0.2 | 0.7 ± 0.2 | 0.3 ± 0.2 | 0.92 | 0.59 | 0.34 |
| **UDCA** | 0.8 ± 0.1 | 1.0 ± 0.5 | 1.3 ± 0.4 | 0.95 | 0.66 | 0.81 |
| **Total** | 1522.3 ± 273.3 | 822.5 ± 171.0 | 778.2 ± 169.0 | 0.07 | 0.06 | 0.99 |
| **Gut luminal bile acids normalized to total bile acids** | | | | | | |
|  | **S-AL** | **S-WM** | **VSG** | **S-AL vs  S-WM** | **S-AL vs VSG** | **S-WM vs VSG** |
| **CA** | 0.0401 ± 0.0094 | 0.0726 ± 0.0316 | 0.0543 ± 0.0247 | 0.65 | 0.92 | 0.86 |
| **CDCA** | ND | 0.0004 ± 0.0004 | ND | - | - | - |
| **DCA** | 0.7502 ± 0.0269 | 0.6394 ± 0.0646 | 0.3599 ± 0.0808 | 0.48 | <0.01 | 0.02 |
| **GCA** | 0.0001 ± 0.0001 | 0.00003 ± 0.00003 | ND | 0.76 | - | - |
| **GDCA** | ND | 0.00003 ± 0.00003 | 0.00007 ± 0.00007 | - | - | 0.86 |
| **HDCA** | 0.0138 ± 0.0048 | 0.0285 ± 0.0063 | 0.0104 ± 0.0018 | 0.11 | 0.87 | 0.04 |
| **LCA** | 0.0121 ± 0.0019 | 0.0151 ± 0.0037 | 0.0129 ± 0.0043 | 0.84 | 0.99 | 0.90 |
| **α-MCA** | 0.1174 ± 0.0066 | 0.1396 ± 0.0257 | 0.3581 ± 0.0648 | 0.93 | <0.01 | <0.01 |
| **β-MCA** | 0.0572 ± 0.0080 | 0.0723 ± 0.0139 | 0.1912 ± 0.0282 | 0.86 | <0.001 | <0.01 |
| **TCA** | 0.0033 ± 0.0011 | 0.0096 ± 0.0038 | 0.0027 ± 0.0012 | 0.23 | 0.98 | 0.15 |
| **TCDCA** | 0.0001 ± 0.0001 | 0.0004 ± 0.0002 | ND | 0.20 | - | - |
| **TDCA** | 0.0009 ± 0.0003 | 0.0019 ± 0.0008 | 0.0005 ± 0.0003 | 0.40 | 0.90 | 0.18 |
| **TMCA (α+β)** | 0.0037 ± 0.0013 | 0.0183 ± 0.0086 | 0.0079 ± 0.0030 | 0.21 | 0.87 | 0.40 |
| **TUDCA** | 0.0005 ± 0.0002 | 0.0007 ± 0.0002 | 0.0004 ± 0.0002 | 0.56 | 0.90 | 0.29 |
| **UDCA** | 0.0006 ± 0.0001 | 0.0011 ± 0.0005 | 0.0018 ± 0.0008 | 0.77 | 0.33 | 0.71 |

Gut luminal bile acid concentrations and abundances from cecal contents. Data presented as mean ± SEM, n=5-6 per group. Data were analyzed using one-factor ANOVA with Tukey’s post hoc analysis. Not detected (ND).

**Supplemental Table S2. Circulating bile acid concentrations and abundances**.

| **Circulating bile acid concentrations (nM)** | | | | | | |  |
| --- | --- | --- | --- | --- | --- | --- | --- |
|  | **S-AL** | **S-WM** | **VSG** | **S-AL vs  S-WM** | **S-AL vs VSG** | **S-WM vs VSG** | |
| **CA** | 394.7 ± 176.7 | 256.7 ± 80.1 | 342.9 ± 148.6 | 0.77 | 0.96 | 0.89 | |
| **CDCA** | 16.2 ± 6.9 | 38.7 ± 12.1 | 19.0 ± 14.3 | 0.42 | 0.99 | 0.48 | |
| **DCA** | 437.9 ± 136.9 | 371.5 ± 79.5 | 563.8 ± 173.0 | 0.94 | 0.80 | 0.57 | |
| **GCA** | 2.1 ± 1.0 | 1.0 ± 0.6 | 3.9 ± 0.2 | 0.49 | 0.14 | 0.01 | |
| **HDCA** | 24.3 ± 8.9 | 41.4 ± 8.2 | 35.6 ± 16.5 | 0.61 | 0.80 | 0.94 | |
| **LCA** | 5.4 ± 1.8 | 6.2 ± 1.6 | 5.3 ± 1.9 | 0.94 | 0.99 | 0.94 | |
| **α-MCA** | 237.1 ± 105.4 | 192.1 ± 41.5 | 311.8 ± 95.9 | 0.93 | 0.81 | 0.56 | |
| **β-MCA** | 151.7 ± 57.6 | 163.7 ± 47.6 | 557.6 ± 292.2 | 0.99 | 0.31 | 0.30 | |
| **TCA** | 412.3 ± 131.6 | 227.0 ± 86.7 | 1006.2 ± 204.1 | 0.68 | 0.04 | <0.01 | |
| **TCDCA** | 16.7 ± 9.1 | 8.8 ± 2.8 | 114.6 ± 46.4 | 0.98 | 0.08 | 0.05 | |
| **TDCA** | 144.1 ± 7.1 | 69.8 ± 12.9 | 166.7 ± 32.2 | 0.08 | 0.75 | 0.02 | |
| **TLCA** | 1.6 ± 0.7 | 0.4 ± 0.4 | 2.5 ± 0.8 | 0.40 | 0.65 | 0.08 | |
| **TMCA (α+β)** | 188.1 ± 29.8 | 155.3 ± 67.2 | 1013.0 ± 203.4 | 0.98 | <0.01 | <0.001 | |
| **TUDCA** | 33.5 ± 3.2 | 27.8 ± 8.6 | 101.4 ± 21.4 | 0.96 | 0.01 | <0.01 | |
| **UDCA** | 31.5 ± 12.3 | 59.7 ± 15.7 | 76.2 ± 56.2 | 0.86 | 0.68 | 0.94 | |
| **Total** | 2097.1 ± 468.6 | 1620.0 ± 371.9 | 4320.6 ± 1117.6 | 0.90 | 0.14 | 0.05 | |
| **Circulating bile acids normalized to total bile acids** | | | | | | |  |
|  | **S-AL** | **S-WM** | **VSG** | **S-AL vs  S-WM** | **S-AL vs VSG** | **S-WM vs VSG** | |
| **CA** | 0.1547 ± 0.0433 | 0.1451 ± 0.0138 | 0.0682 ± 0.0174 | 0.96 | 0.08 | 0.11 | |
| **CDCA** | 0.0065 ± 0.0017 | 0.0252 ±0.0058 | 0.0032 ± 0.0019 | 0.01 | 0.81 | <0.01 | |
| **DCA** | 0.2003 ± 0.0385 | 0.2580 ± 0.0420 | 0.1312 ± 0.0281 | 0.53 | 0.41 | 0.06 | |
| **GCA** | 0.0012 ± 0.0006 | 0.0004 ± 0.0002 | 0.0011 ± 0.0002 | 0.29 | 0.99 | 0.33 | |
| **HDCA** | 0.0137 ± 0.0073 | 0.0273 ± 0.0035 | 0.0071 ± 0.0014 | 0.11 | 0.56 | 0.01 | |
| **LCA** | 0.0024 ± 0.0007 | 0.0039 ± 0.0010 | 0.0012 ± 0.0003 | 0.35 | 0.47 | 0.04 | |
| **α-MCA** | 0.0933 ± 0.0269 | 0.1225 ± 0.0253 | 0.0719 ± 0.0129 | 0.64 | 0.78 | 0.25 | |
| **β-MCA** | 0.0650 ± 0.0135 | 0.0972 ± 0.0127 | 0.1061 ± 0.0337 | 0.61 | 0.46 | 0.96 | |
| **TCA** | 0.2320 ± 0.0837 | 0.1246 ± 0.0273 | 0.2471 ± 0.0286 | 0.31 | 0.98 | 0.19 | |
| **TCDCA** | 0.0097 ± 0.0051 | 0.0063 ± 0.0017 | 0.0273 ± 0.0105 | 0.94 | 0.23 | 0.11 | |
| **TDCA** | 0.0836 ± 0.0171 | 0.0506 ± 0.0114 | 0.0477 ± 0.0128 | 0.25 | 0.19 | 0.99 | |
| **TLCA** | 0.0008 ± 0.0004 | 0.0002 ± 0.0002 | 0.0007 ± 0.0003 | 0.31 | 0.94 | 0.45 | |
| **TMCA(α+β)** | 0.1032 ± 0.0267 | 0.0831 ± 0.0177 | 0.2485 ± 0.0240 | 0.82 | <0.01 | <0.001 | |
| **TUDCA** | 0.0209 ± 0.0058 | 0.0171 ± 0.0031 | 0.0272 ± 0.0074 | 0.89 | 0.73 | 0.42 | |
| **UDCA** | 0.0127 ± 0.0027 | 0.0385 ± 0.0069 | 0.0116 ± 0.0057 | 0.02 | 0.99 | 0.01 | |

Circulating bile acids concentrations and abundances from serum. Data presented as mean ± SEM, n=5-6 per group. Data were analyzed using one-factor ANOVA with Tukey’s post hoc analysis.

Cholic Acid (CA), Chenodeoxycholic Acid (CDCA), Deoxycholic Acid (DCA), Glycocholic Acid (GCA), Hyodeoxycholic Acid (HDCA), Lithocholic Acid (LCA), Alpha-Muricholic Acid (α-MCA), Beta-Muricholic Acid (β-MCA), Taurocholic Acid (TCA), Taurochenodeoxycholic Acid (TCDCA), Taurodeoxycholic Acid (TDCA), Taurolithocholic Acid (TLCA), Tauroursodeoxycholic Acid (Alpha and Beta combined) (TMCA (α+β)), Tauroursodeoxycholic Acid (TUDCA), Ursodeoxycholic Acid (UDCA).
